# Crosstalk between Bcl-2 family and Ras family small GTPases: potential cell fate regulation?

**DOI:** 10.3389/fonc.2012.00206

**Published:** 2013-01-02

**Authors:** Jia Kang, Shazib Pervaiz

**Affiliations:** ^1^ROS, Apoptosis and Cancer Biology Laboratory, Department of Physiology, Yong Loo Lin School of MedicineSingapore, Singapore; ^2^NUS Graduate School for Integrative Sciences and Engineering, National University of SingaporeSingapore, Singapore; ^3^Cancer and Stem Cell Biology Program, Duke-NUS Graduate Medical SchoolSingapore, Singapore; ^4^Singapore-Massachusetts Institute of Technology AllianceSingapore, Singapore

**Keywords:** BCL-2 family, Ras family small GTPases, apoptosis, interaction, redox

## Abstract

Cell fate regulation is a function of diverse cell signaling pathways that promote cell survival and or inhibit cell death execution. In this regard, the role of the Bcl-2 family in maintaining a tight balance between cell death and cell proliferation has been extensively studied. The conventional dogma links cell fate regulation by the Bcl-2 family to its effect on mitochondrial permeabilization and apoptosis amplification. However, recent evidence provide a novel mechanism for death regulation by the Bcl-2 family via modulating cellular redox metabolism. For example overexpression of Bcl-2 has been shown to contribute to a pro-oxidant intracellular milieu and down-regulation of cellular superoxide levels enhanced death sensitivity of Bcl-2 overexpressing cells. Interestingly, gene knockdown of the small GTPase Rac1 or pharmacological inhibition of its activity also reverted death phenotype in Bcl-2 expressing cells. This appears to be a function of an interaction between Bcl-2 and Rac1. Similar functional associations have been described between the Bcl-2 family and other members of the Ras superfamily. These interactions at the mitochondria provide novel opportunities for strategic therapeutic targeting of drug-resistant cancers.

## INTRODUCTION

Despite the complexity of the various commands and control pathways implicated in oncogenic transformation of cells of different origins, the common denominator in all forms of neoplasia is the dysregulated or defective ratio between cell proliferation and cell death (Hanahan and Weinberg, 2011). Any disturbance of this ratio due to either enhanced proliferation signals or defective death circuits would result in an abnormal accumulation of cells, thereby leading to carcinogenesis. To that end, there is strong evidence that an altered cellular metabolism fuels the process of transformation by creating an intracellular milieu conducive for cell survival and growth. Indeed, recent observations have underscored the critical role that cells’ metabolic processes play in oncogene-driven carcinogenesis, such as Ras, phosphoinositide 3-kinase (PI3K)/Akt, myc, and others (Shields et al., 2011; Herranz et al., 2012; Ho et al., 2012; Murugan et al., 2012; Zubova et al., 2012). These studies highlight the differences in the metabolic demands and bio-energetic wiring of cancer cells and non-cancerous cells. There is indeed a shift in the steady-state cellular and mitochondrial redox milieu of cancer cells toward a slight pro-oxidant state that promotes cell survival by enhancing proliferative signals and cell cycle progression, but at the same time inhibiting death execution. In this sense, an altered redox status has been implicated in the transformation caused by the Ras oncogene activation following an increase in Nox activity mediated by Rac (Irani et al., 1997; Irani and Goldschmidt-Clermont, 1998) as well as in the regulation of apoptosis by the anti-apoptotic protein Bcl-2, the prototypic member of the Bcl-2 family. Not only do members belonging to these two families of proteins (Ras and Bcl-2) elicit similar functional outcomes, there is also evidence for a direct and/or indirect crosstalk between specific proteins from these families. In this regard, there are reports of protein–protein interactions as well as co-localization of member proteins. Here we attempt to present a brief review of the crosstalk between the Bcl-2 family members and the small guanosine triphosphatases (GTPases) of the Ras superfamily, which also modulate the pro- and anti-apoptotic properties of Bcl-2 members.

## PRO- AND ANTI-APOPTOTIC Bcl-2 FAMILY MEMBERS

The Bcl-2 family of proteins is made of three subgroups according to the Bcl-2 homology (BH) domains they contain and their pro- or anti-apoptotic activities. Members that contain all four BH domains (BH1, BH2, BH3, and BH4), which include Bcl-2, Bcl-xL, Bcl-w, Mcl-1, and A1, are classified under the anti-apoptotic or pro-survival category (Low et al., 2011). Overexpression of any of these members blocks apoptosis execution; while genetic knockdown reveals their essential roles in cell survival. Members that contain BH1, BH2, and BH3 domains but not BH4 domain such as Bax, Bak, and Bok are classified under pro-apoptotic category. Bax and Bak are ubiquitously expressed in various tissues while Bok is mainly present in the reproductive organs. There is a third divergent class of pro-apoptotic members that only share sequence homology in the BH3 domain. These proteins are called BH3-only proteins and consist of Bad, Bid, Bim, Bmf, BNIP3, Hrk, Noxa, and PUMA (Strasser, 2005; Youle and Strasser, 2008).

As regulators of apoptosis, the Bcl-2 proteins will dictate whether the cell lives or dies, depending upon the proportion of pro- and anti-apoptotic components. In response to stress signals, such as exposure to radiation, hypoxia, deprivation of nutrients, heat shock, viral infection, and DNA damage, the pro-apoptotic members are activated, resulting in their translocation to and oligomerization at the mitochondria. Such oligomerization results in mitochondrial outer membrane permeabilization (MOMP), thereby facilitating the release of apoptogenic factors such as cytochrome C, Smac/DIABLO, and apoptosis-inducing factor (AIF) from the mitochondria. This is the classical type II or the intrinsic pathway of apoptosis, a genetically programmed process with an orchestrated series of events leading to the death of a cell. However, in the event of overexpression of the anti-apoptotic proteins, which is invariably observed in drug-resistant cancers, the pro-apoptotic activity of Bax, Bak, or Bok is neutralized by the formation of homo- and heterodimers that prevent oligomerization of the pro-apoptotic members (Johnstone et al., 2002). The BH3-only proteins, on the other hand, can act as either antagonists of anti-apoptotic members or direct activators of pro-apoptotic members.

The elucidation of the 3-D structure of human Bcl-xL revealed a pore-forming protein (Muchmore et al., 1996), and subsequently other members such as Bcl-2 and Bax were shown to be capable of forming pores in artificial membranes (Antonsson et al., 1997; Minn et al., 1997; Schendel et al., 1997). It was not until 2001 that the 3D structure of Bcl-2 was resolved with its unstructured loop region being replaced by that of Bcl-xL. Although both proteins share a similar overall helical fold and function, they differ in the highly flexible unstructured loop region, which contributes to their different solubilities (Petros et al., 2001). Another key structural difference lies in the amino acid residues and the size of the hydrophobic groove formed by the BH1, BH2, and BH3 domains, which is the important interaction site with pro-apoptotic members such as Bax and Bak (Sattler et al., 1997) and this probably explains the different binding affinities of Bcl-2 and Bcl-xL toward them. The BH3 domain is critical in the functions of the Bcl-2 family proteins, because not only is this domain of Bcl-2 responsible for interacting with and antagonizing the pro-apoptotic members but also is the domain used by the pro-apoptotic Bax and Bak to antagonize Bcl-2-mediated protection against apoptosis. Deletion of this domain from Bax and Bak results in impairment of the pro-apoptotic activity and binding toward Bcl-2 and Bcl-xL, while transfection of this domain alone can lead to apoptosis, similar to BH3-only proteins.

## NETWORKS OF Bcl-2 FAMILY PROTEINS WITH NON-HOMOLOGOUS PARTNERS FOR APOPTOSIS MODULATION: A FOCUS ON SMALL GTPases OF THE Ras FAMILY

As mentioned above, the homologous interaction within the Bcl-2 family are responsible for the functional outcomes in terms of apoptosis induction and its regulation. However, many other non-homologous proteins directly or indirectly associate with Bcl-2 family members, thereby modulating their pro- or anti-apoptotic properties. Thus, the Ras and Rac small GTPases modulate cell fate decisions by interacting with Bcl-2, as will be discussed in this review.

### Ras SUPERFAMILY OF SMALL GTPases: STRUCTURES AND FUNCTIONS

The Ras superfamily of small GTPases are monomeric G proteins with molecular mass ranging from 20 to 30 kDa. To date, more than 100 proteins have been included in this superfamily, which is further subdivided into eight groups based on amino acid sequence, protein structure and function similarities: Ras, Rad, Rab, Rap, Ran, Rho, Rheb, Rit, and Arf (Wennerberg et al., 2005). These small GTPases function as binary molecular switches, whose activity is regulated through the binding, hydrolysis, and release of GTP. The GTP/GDP cycling is in turn mediated by the joint activities of a series of guanine nucleotide exchange factors (GEFs) and GTPase-activating proteins (GAPs). A set of G box GDP/GTP-binding motif elements make up the G domain, which is conserved both structurally and functionally across all Ras superfamily members as well as other GTPases.

Upon triggered by various signals captured by surface receptors (Etienne-Manneville and Hall, 2002; Welch et al., 2003), GEFs promote the activation of small GTPases (Schmidt and Hall, 2002; Rossman et al., 2005). The interaction of GEFs with the small GTPases destabilizes binding of the nucleotide and results in the formation of a nucleotide free intermediate. GTP is more concentrated intracellularly than GDP, and thus saturates small GTPases more readily. The change in bound nucleotide from GDP to GTP alters the conformation in the Switch 1 and Switch 2 regions allowing the small GTPases to bind downstream effector proteins, e.g., scaffold proteins (such as p67phox and IQGAPs), serine/threonine kinases [such as mitogen-activated protein kinase kinase kinase (MEKKs) and p21-activated kinases (PAKs)], lipid kinases (such as PI3K) and lipases [such as phospholipase D (PLD), and PLC-β2], etc., (Bishop and Hall, 2000). Following transient activation of downstream pathways, GTP is hydrolyzed to by GAPs which complement the slow intrinsic activity of small GTPases (Bishop and Hall, 2000). The signaling specificity (i.e., activation of specific small GTPase pathway) is governed in part by GEFs since the specificity of GEFs toward the GTPases varies. For example in the Rho subfamily, Tiam1 is a specific activator of Rac1 (Hordijk et al., 1997) while Vav promiscuously bind RhoA, RhoG, Rac1, and Cdc42 (Han et al., 1997). A third class of proteins named guanine nucleotide dissociation inhibitors (GDIs) regulate the activities of Rho and Rab subfamilies. GDIs mask the C-terminal lipid moieties and sequester Rho and Rab GTPases in a soluble state in the cytosol (Konstantinopoulos et al., 2007).

The post-translational addition of the lipid moiety is called prenylation, which is important for membrane associations and subcellular targeting. The majority of Ras and Rho subfamily members contain a CAAX tetrapeptide motif (where C denotes cysteine, A represents any prenylated amino acid and X may be any amino acid) at the C-terminal which could be either farnesylated (covalent addition of farnesyl pyrophosphate) as in the case of Ras or geranylgeranylated (addition of geranylgeranyl pyrophosphate) as for the RhoA, Rac1 and Cdc42 GTPases. K-Ras and N-Ras isoforms could undergo alternative geranylgeranylation as well when the farnesylation process is inhibited (Berndt et al., 2011). Apart from prenylation, certain small GTPases such as Rac1 and Ras could also undergo palmitoylation where the cysteine residues immediately upstream of the CAAX motif are reversibly modified by the fatty acid palmitate (Laude and Prior, 2008; Navarro-Lerida et al., 2012). Yet other small GTPases like some members of the Arf subfamily are modified by myristoylation at the N-terminal. All these modifications, together with the conserved polybasic region (comprised of multiple lysines or arginines) at the C-terminal, facilitate association of the small GTPases with various membrane compartments to exert distinct biological functions (Wennerberg et al., 2005).

### IMPACT OF Ras SUPERFAMILY OF SMALL GTPases ON APOPTOTIC SIGNALING

Ras superfamily of small GTPases are involved in a plethora of intracellular functions including cytoskeleton organization, gene transcription, reactive oxygen species (ROS) production, cell cycle progression, apoptotic regulation, differentiation, to name just a few. Here we present a brief overview of the role that Ras and the Rho subfamily member Rac play in the regulation of apoptotic cell death, and interestingly the crosstalk between these proteins and the Bcl-2 family in determining the functional outcome.

#### Ras

Activating mutations in the Ras oncoproteins is encountered in about 30% of human malignancies (McCubrey et al., 2012b; Sacco et al., 2012). The transforming ability of Ras proteins suggests that Ras-regulated pathways could either promote cell proliferation and/or inhibit cell death, particularly apoptotic execution. Although considerable evidence has supported this, there are also many other reports that demonstrate the opposite where Ras can inhibit proliferation and lead to apoptosis, indicating a strong dependency on cell type and cellular context.

The best characterized Ras effectors are the Raf family of serine/threonine kinases which include A-Raf, B-Raf, and Raf-1. Upon interaction with the effectors, downstream mitogen-activated protein (MAP) kinase kinases (MEKs) are activated, which in turn activate the MAP kinases extracellular signal-regulated kinases (ERKs) leading to phosphorylation of downstream targets that either positively or negatively influence apoptosis. Regulation of the Raf/MEK/ERK pathways in apoptosis is partly due to the post-translational phosphorylation of Bcl-2 family members including Bad, Bim, Mcl-1, and more controversially Bcl-2 (McCubrey et al., 2007). For example, Bad is known to be phosphorylated on Ser112, leading to its inactivation and subsequent sequestration by 14-3-3 proteins. This releases Bcl-2 and Bcl-xL to carry out their anti-apoptotic function (Zha et al., 1996). Similarly, phosphorylation of Bim displaces it from Bcl-2, Bcl-xL, and Mcl-1 followed by its ubiquitination and proteosomal degradation (Ley et al., 2003; Luciano et al., 2003; Weston et al., 2003; Harada et al., 2004). In this same pathway, Bcl-2 was also reported to be phosphorylated on critical residues within the flexible loop, resulting in enhanced anti-apoptotic activity (Deng et al., 2000, 2001). In breast cancer cells, ectopic expression of Raf could increase the protein levels of Bcl-2, which is likely to be due to the enhanced phosphorylation of downstream transcription factors that bind to the promoter region of Bcl-2 upon activation of the pathway (Weinstein-Oppenheimer et al., 2001; Davis et al., 2003). Moreover, apart from activating downstream MEK/ERK, Raf-1 was also shown to regulate apoptosis at the mitochondrial membrane by phosphorylating Bad resulting in its release into the cytosol (Wang et al., 1996; Salomoni et al., 1998; Neshat et al., 2000; Hindley and Kolch, 2002).

In addition to Raf kinases, PI3K is another Ras effector, the activation of which results in stimulation of the activity of the serine/threonine kinase Akt, an event usually associated with apoptotic evasion (Markman et al., 2012). Similarly to the Raf/MEK/ERK pathway, Akt can also phosphorylate Bim at Ser87 promoting its sequestration by 14-3-3 proteins (Qi et al., 2006). In addition, Akt phosphorylation of the transcription factor Foxo3A would suppress its ability to induce the transcription of the BH3-only protein Puma, which induces apoptosis through interactions with Bax/Bak and Mcl-1 (McCubrey et al., 2007). However, crosstalks between these two pathways complicate the whole picture. While Raf/MEK/ERK pathway is usually associated with proliferation and drug resistance in cells of hematopoietic lineage, mutations in the phosphatase and tensin homolog (PTEN) that result in hyper-activated Akt in certain prostate cancer cell lines would lead to suppression of this pathway. Actually, evidence has accumulated over the past two decade pointing to the paradoxical role of Ras in inducing apoptosis in situations where cancer cells are subjected to adverse environmental conditions or apoptotic stimuli or in the case of ectopic expression of constitutively active Ras. One contributing factor is the association of Ras with Bcl-2 family. Ras was indeed clearly shown to up-regulate a pro-apoptotic Bcl-2 family member, BNIP3. Nitric oxide exposure in a mouse leukemia cell line with macrophage characteristics triggers activation of the transcription factor, hypoxia-inducible factor 1 (HIF-1) mediated through ERK, which then binds to the BNIP3 promoter, leading to apoptosis (An et al., 2006). A direct association of Ras with Bcl-2 (Chen and Faller, 1996; Denis et al., 2003) or Bcl-xL (Bivona et al., 2006) has also been demonstrated. Another argument in favor of Ras-mediated apoptosis is that its downstream small GTPase Rac could associate with Ras through its associated GEF Tiam1, thereby linking the activation of Rac with that of Ras. The detailed mechanisms of these pathways are discussed in the following sections.

Generally speaking, the decision to turn on either pro-survival or pro-death pathways upon Ras activation depends very much on the environmental cues (e.g., growth factors and extracellular matrix interactions), predominant isoform(s) of Ras expressed in a particular cell type (Choi et al., 2004; Ninomiya et al., 2004), variations in the expression levels of Ras effectors as well as the differential subcellular localizations of Ras resulting from distinct post-translational modifications (Overmeyer and Maltese, 2011). For example, treating N-Ras or K-Ras expressing cells with a farnesyltransferase inhibitor could turn off the pro-survival pathway and switch on the pro-apoptotic one which is probably an effect of alternative geranylgeranylation on the subcellular compartmentalization and/or effector interactions of Ras (Geryk-Hall et al., 2010). Apart from prenylation, it is reported that phosphorylation on Ser181 of Ras in the polybasic region by protein kinase C (PKC) would stimulate apoptosis possibly due to the shuttling of Ras from plasma membrane to intracellular compartments such as the mitochondria where it could interact with Bcl-2 family members as mentioned earlier (Bivona et al., 2006).

#### Rac

Ras-related C3 botulinum toxin substrates, or commonly known as Rac, belongs to the Rho (Ras homologs) subfamily. Rac1, was first identified together with Rac2 in 1989, which bears 58% homology to Rho proteins and 20–30% homology to Ras proteins (Didsbury et al., 1989). Subsequently, other forms of Rac proteins, namely Rac3 and Rac1b were discovered. The tissue distribution varies among different forms, with Rac1 ubiquitously expressed in most tissues, Rac2 mainly in hematopoietic cells, Rac3 highly enriched in the brain but expressed in other tissues as well at lower levels and the splice variant Rac1b minimally expressed in normal cells but highly enriched in cancers such as breast and colorectal cancers (Haataja et al., 1997; Jordan et al., 1999; Schnelzer et al., 2000; Chan et al., 2005).

The regulatory role of Rac in apoptosis is also somewhat controversial and could be due to the distinct effectors in different cellular contexts. For example, Rac1 could induce mitogenic signals via activation of ERK1/2, JNK, PI3K, and Akt (Aznar and Lacal, 2001; Kwei et al., 2006). In contrast to ERK and Akt activation, JNK activation could result in phosphorylation of Bim at Ser65 leading to apoptotic induction by promoting Bax homodimerization (Lei and Davis, 2003; Putcha et al., 2003). JNK could also phosphorylate 14-3-3 proteins allowing translocation of Bax from the cytosol to the mitochondria membrane to promote apoptosis (Okuno et al., 2004).

The ROS producing ability of Rac through Nox and the controversial impact of ROS on apoptotic signaling depending on the amount and specific species produced, could also explain why Rac activation results in either pro-survival or pro-apoptotic cell fate. One of the first identified effector proteins of Rac was p67phox, a subunit of the NADPH oxidase complex (Diekmann et al., 1994). Active GTP-bound Rac binds to cytosolic p67phox (associated with p47phox and p40phox) and then recruits the protein complex to the membranes where they bind to the integral membrane components gp91phox (Nox2) and p22phox for the assembly and activation of the multimolecular NADPH oxidase to produce ${\text{O}}_{2}^{-}$ (Babior, 1999; Dinauer, 2003; Groemping and Rittinger, 2005). In addition to the binding of Rac and gp91phox at the membranes has also been demonstrated to be essential in activation of electron transport through the heterodimeric flavocytochrome b_558_ consisting of gp91phox and p22phox (Nisimoto et al., 1999). Based on site-directed/deletional mutagenesis and peptide walking studies, regions in Rac critical for NADPH oxidase activation lie in the Switch 1 effector loop, the insert domain as well as the C-terminal basic motif (Kwong et al., 1993; Diekmann et al., 1994; Joseph and Pick, 1995; Freeman et al., 1996; Hirshberg et al., 1997). Although this activation model was first described for phagocytic isoform Nox2-based NADPH oxidase, Rac has been shown to activate Nox1- and Nox3-based complexes in non-phagocytic cells as well (Sundaresan et al., 1996; Ueyama et al., 2006) and the modulatory activity is achieved through Rac-binding proteins Noxa1 (homolog of p67phox) and Noxo1 (homolog of p47phox; Bokoch and Diebold, 2002; Cheng and Lambeth, 2004). A few reports have linked ROS production mediated by Rac activation to apoptosis (Overmeyer and Maltese, 2011), while other work supports the opposite view whereby Rac1 activation is pro-survival by virtue of the resultant “pro-oxidant” intracellular milieu. The mitogenic activity of a mild but chronic elevation could alter the activities of a plethora of intracellular signaling targets: activation of transcription factors, oxidative inhibition of phosphatases and modulation of protein kinases (Sauer et al., 2001), which in turn switch on the downstream mediators of proliferation. Indeed, as a downstream target for Ras, Rac1 activation and ROS production were shown to contribute to Ras-induced mitogenic signaling in fibroblasts (Irani et al., 1997). The oncogenic potential of Rac1, mediated through ROS production, was highlighted in an earlier report where we showed that a constitutively active mutant of Rac1, namely V12, increased intracellular ${\text{O}}_{2}^{-}$ production in human melanoma M14 cells leading to chemoresistance, while transient introduction of the dominant negative mutant N17 decreased ${\text{O}}_{2}^{-}$ levels and enhanced apoptosis sensitivity. In addition, inhibition of Rac1 in T24 bladder carcinoma cells expressing mutant Ras also significantly decreased ${\text{O}}_{2}^{-}$ levels and increased their sensitivity to both receptor- and drug-induced apoptosis. On the contrary, the effect could be reversed with inhibition of the cytosolic ${\text{O}}_{2}^{-}$ scavenger Cu/ZnSOD, thus indicating that the apoptotic resistance of oncogenic Ras-expressing cancer cells could be associated with an increase in steady-state intracellular ${\text{O}}_{2}^{-}$ mediated through Rac1 activation (Pervaiz et al., 2001).

### INTERACTIONS OF Ras, Raf-1, AND Rac1 WITH Bcl-2 OR Bcl-xL TO MODULATE THEIR ANTI-APOPTOTIC PROPERTIES

#### Ras

In lymphocytes, activated H-Ras can trigger Fas-mediated apoptosis which is inhibited through increased interaction of the BH4 domain of Bcl-2 with mitochondrial Ras. To that end, the CAAX motif of Ras required for farnesylation is demonstrated to be essential for its apoptotic signaling and Bcl-2 association. In addition, increased phosphorylation of Bcl-2 is observed with H-Ras activation. Prevention of the phosphorylation and decreasing Bcl-2’s association with Ras could sensitize cells to apoptosis (Denis et al., 2003). In another study, a somewhat different picture is presented where the isoform K-Ras is found to associate with Bcl-xL at the mitochondria. Phosphorylation of K-RasB at the polybasic region reduces the net positive charge and weakens its association with the plasma membrane. The electrostatic switch of K-RasB thus results in its mitochondrial translocation neutralizing the anti-apoptotic function of Bcl-xL (Bivona et al., 2006).

#### Raf-1

Raf-1, a signal transducing serine/threonine kinase in the Ras pathway has also been shown to interact with Bcl-2 and inhibit apoptosis (Wang et al., 1996). Upon phosphorylation by PAK1, Raf-1 is targeted to the mitochondria through its interaction with the BH4 domain of Bcl-2. Mitochondrial Raf-1 then phosphorylates Bad and releases Bcl-2 from Bad–Bcl-2 complex, promoting cell survival (Jin et al., 2005).

#### Rac1

Similarly to Ras and Raf-1, we recently reported that Rac1 may also be found at the mitochondrial membrane where it interacts with Bcl-2. The BH3 domain and the adjacent flexible loop region of Bcl-2 are involved in this interaction (Velaithan et al., 2011). It is proposed that this interaction stabilizes Bcl-2’s anti-apoptotic activity through promotion of the pro-oxidant intracellular milieu since transient transfection of the dominant negative mutant Rac1N17 resulted in a decrease in ${\text{O}}_{2}^{-}$ levels and an increase in the sensitivity of Bcl-2-overexpressing chronic myeloid leukemia (CEM) cells to receptor or drug-induced apoptosis (Clement et al., 2003). Similar findings are observed with synthetic Bcl-2 BH3 domain peptides that disrupt the interaction or siRNA-mediated silencing of Rac1 expression or a pharmacological inhibitor of Rac1 (Velaithan et al., 2011). Interestingly, Rac2 instead of Rac1, is implicated in the survival pathway of Bcl-xL by increasing the expression levels of Bcl-xL and decreasing the expression levels of Bad (Yang et al., 2000). In addition, overexpression of Bcl-xL could rescue the effects seen with Rac2 deficiency (Mizukawa et al., 2011). However, the existence of a physical interaction between Rac2 and Bcl-xL is yet to be explored.

## SIGNIFICANCE OF THE Bcl-2–Ras FAMILY CROSSTALKS IN DRUG-RESISTANT CANCERS

One of the main challenges for cancer therapeutic management is drug resistance, which could be contributed by several mechanisms including target modification, drug inactivation, drug extrusion, and apoptotic execution inhibition. Studies to understand the molecular mechanisms governing chemotherapeutic drug resistance show that both the Bcl-2 and Ras family members are implicated either because of overexpression (as for anti-apoptotic Bcl-2 family members; Reed, 1995; Nuessler et al., 1999; Thomas et al., 2013; Zhang et al., 2012) or of ectopic mutational activation (as for Ras, Raf; Weinstein-Oppenheimer et al., 2001; McCubrey et al., 2012a). **Table 1** lists some of the examples of drug-resistant cancers or immortalized cells due to abnormally regulated Bcl-2 and Ras pathways that act in concert. In MCF-7 breast cancer cells, overexpression of the constitutively active Raf-1 resulted in resistance toward doxorubicin. Induction of Raf-1 activity led to increased Bcl-2 expression and a further overexpression of Bcl-2 resulted in greater resistance (Davis et al., 2003). In another study done in oncogene v-Ha-*ras*-transformed NIH/3T3 cells, marked resistance toward alkylating agents such as methylmethane sulfonate (MMS) was observed, which could be partially explained by the constitutively elevated Bcl-2 protein levels in *ras*-transformed cells as compared to parental cells (Kuo et al., 1997). Crosstalk between another isoform of Ras, c-K-Ras and the pro-apoptotic Bcl-2 family member Bax is reported as well where resistance to sulindac sulfide, a non-steroidal anti-inflammatory drug from the arylalkanoic acid class, was observed following *ras*-transformation which could probably be mediated through specific down-regulation of Bax expression (Arber et al., 1997). Furthermore, concurrent involvement of both Ras and Bcl-2 pathways is observed in various other drug-resistant cancer models, such as imatinib-resistant acute lymphoblastic leukemia with Philadelphia chromosome (Ph^+^ ALL; Suzuki et al., 2010), both cisplatin- and paclitaxel-resistant ovarian cancer (Wang et al., 2010) as well as VP-16- and cisplatin-resistant prostate cancer (Sinha et al., 1995). However, the exact underlying molecular mechanisms of the crosstalks were not covered in those studies. Further investigation on how the crosstalks between the two families lead to drug resistance may lay a foundation for designing adjuvant therapies aiming at improving the success rate for many clinically available chemotherapeutic drugs.

**Table 1:** Bcl-2 and Ras family members’ crosstalks in drug-resistant cancers and immortalized cells

Cancer types	Resistant to	Abnormal genetic/epigenetic events involved	Reference
Breast cancer	Doxorubicin; paclitaxel	Ectopic activation of Raf-1 that led to increased Bcl-2 expression	Davis et al. (2003)
v-Ha-*ras*-transformed NIH/3T3 cells	Methylmethane sulfonate (MMS)	Constitutively elevated Bcl-2 levels upon *ras*-transformation	Kuo et al. (1997)
Immortalized rat enterocytes	Sulindac	Mutant K-Ras-mediated transformation led to resistance which might result from specific down-regulation of Bax expression	Arber et al. (1997)
Philadelphia chromosome-positive acute lymphoblastic leukemia (Ph^+^ ALL)	Imatinib	Concurrent increase in the activation of Ras, phosphorylation of MEK and ERK, and expression of Bcl-2	Suzuki et al. (2010)
Ovarian cancer	Cisplatin; paclitaxel	Autocrine production of IL-6-mediated resistance is associated with increased expression of Bcl-2 and Bcl-xL as well as activation of Ras/MEK/ERK and PI3K/Akt pathways	Wang et al. (2010)
Prostate cancer	VP-16; cisplatin	Resistant cells harbor both Bcl-2 protein overexpression and H-Ras mRNA overexpression	Sinha et al. (1995)
Non-small cell lung cancer (NSCLC)	Gefitinib	Combination treatment of gefitinib and lovastatin led to down-regulation of Bcl-2 and up-regulation of Bax in cells with mutant K-Ras	Park et al. (2010)

## FUTURE PERSPECTIVES

**FIGURE 1:**
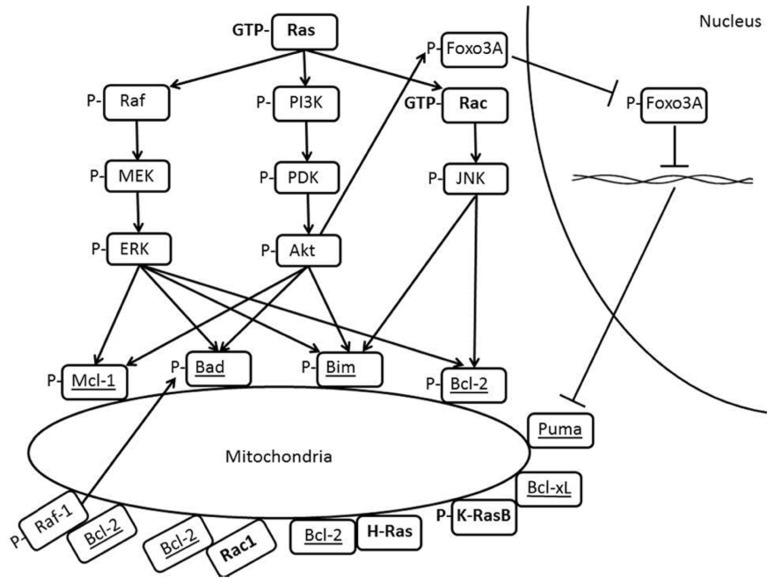
**Crosstalks between Ras and Bcl-2 family members**.

Bcl-2 family proteins are well-known regulators of apoptosis by virtue of their abilities to either promote (for pro-apoptotic members) or prevent (for anti-apoptotic members) the outer membrane permeabilization through homologous interactions within the family. Recently, an alternative paradigm has surfaced where by overexpression of Bcl-2 confers survival advantage to cancer cells by creating a pro-oxidant milieu. It should be stressed that the Ras superfamily of small GTPases, comprising more than 100 members, is most diverse and versatile in signal transducing capabilities. The founding member Ras and Rac, a member of the Rho subfamily, are implicated in anti-apoptotic signaling, although controversial reports have demonstrated the paradoxical role of both proteins in cell fate decision. The intriguing findings on the associations between Ras GTPases or effectors in the pathway like Raf-1 and Bcl-2 family members, be it direct physical interaction or indirect correlation as summarized in **Figure 1**, underscores the contrasting effects of Ras family members in promoting cell survival or cell death. In addition, the converging role of Rac1 and Bcl-2 in promoting the pro-oxidant state of cancer cells through physical interaction opens up a new horizon for future redox-based therapeutic designs.

## Conflict of Interest Statement

The authors declare that the research was conducted in the absence of any commercial or financial relationships that could be construed as a potential conflict of interest.
